# Outcomes of Corneal Tattooing by Rotring Painting Ink in Disfiguring Corneal Opacities

**DOI:** 10.1155/2018/5971290

**Published:** 2018-06-25

**Authors:** Alahmady H. Alsmman, Engy Mohamed Mostafa, Amr Mounir, Mahmoud Mohamed Farouk, Mohamed Gamal Elghobaier, Gamal Radwan

**Affiliations:** ^1^Department of Ophthalmology, Sohag Faculty of Medicine, Sohag University, Sohag 82524, Egypt; ^2^Egyptian Police Hospitals, Cairo, Egypt

## Abstract

**Aim:**

To evaluate corneal tattooing with Rotring painting ink (Rotring Ink, Hamburg, Germany) as an available and affordable surgical technique to improve cosmetic appearance in the eyes with disfiguring corneal opacities.

**Methods:**

Fifty-three blind eyes with corneal disfiguring opacities underwent corneal tattooing using Rotring painting ink (Rotring Ink, Hamburg, Germany) by multiple transepithelial intrastromal injections under topical anesthesia. Complete ophthalmic examination and ocular ultrasonography were performed, and photographs of the patients' eyes were taken. Follow-up period was at least 12 months.

**Results:**

On the first postoperative day, all patients presented with mild conjunctival injection and foreign body sensation. After the end of the follow-up period, 51 patients (96%) were satisfied of cosmetic appearance while only 2 patients (4%) post-op cosmetic results were less than their expectations; however, they were better in appearance. No major complications like corneal erosions; corneal ulcers or corneal melting was noted in any case.

**Conclusions:**

Corneal tattooing with Rotring painting ink in blind disfigured eyes achieves favourable cosmetic results and is associated with high patient satisfaction. With better case selection, a high post-op satisfaction was achieved. Corneal tattooing acts as an alternative to more sophisticated and expensive cosmetic reconstructive surgery. This trial is registered with ISRCTN46626979.

## 1. Introduction

Corneal tattooing is as old as Galen (131-210 A.D.) who is thought to be the first to use reduced copper sulphate to mask a corneal leukoma [1, 2]. The literature also mentions von Weckerin [3] back in 1870 who used India ink into a scarred cornea. Yet, the role of corneal tattooing is not limited to improving the cosmetic appearance of patients but also to improving the visual quality in situations as aniridia, albinism, large colobomas, and large peripheral iridotomy [4, 5].

Corneal tattooing is one of the options offered to patients with corneal opacities as well as tinted contact lenses, enucleation, or evisceration with orbital prosthesis and the more popularly penetrating keratoplasty [6]. The availability of other options affected the popularity of corneal tattooing [7]. Various tattooing methods were used such as chemical dyeing with the use of gold or platinum chloride [8] and nonmetallic tattooing with India ink, China ink, lamp black, and other organic dyes [9].

Introducing corneal tattooing to the cornea was carried out by several maneuvers including simple corneal staining, anterior stromal needle puncture, or corneal stromal pigment insertion through lamellar intrastromal channels [10]. More recently, excimer laser and lamellar corneal cuts by femtolaser were used [11].

In our study, we aimed at investigating the safety and durability of corneal tattooing by Rotring painting ink in blind disfigured eyes as an alternative to the more expensive and invasive cosmetic option.

## 2. Patients and Methods

Fifty-three consecutive, nonrandomized eyes of 53 blind patients with total corneal leukomas were enrolled in this prospective, interventional, noncomparative clinical study conducted at the Ophthalmology Department of Sohag University Hospital, Egypt, from September 2015 to April 2016. Causes of blindness and corneal leukoma are stated in Table 1. All patients were seeking medical advice to improve their cosmetic appearance. All of them either did not tolerate the tinted contact lens or were not comfortable using them. Penetrating keratoplasty could not be considered as an alternative due to the blind eye, the high risk of complications along with the high cost involved. Ophthalmic examination was performed thoroughly including slit-lamp biomicroscopy to assess the depth of corneal opacity, corneal thickness, and corneal vascularization. Ocular ultrasonography was also part of the examination to exclude atrophia bulbi or intraocular tumour. Inclusion criteria were superficial or deep corneal opacities causing severe disfigurement in the blind eyes. All patients have dark brown-colored iris of the fellow healthy eye. Exclusion criteria included chronically inflamed eyes, severe corneal calcification or neovascularization, phthisical eyes, anterior staphyloma, and patients of high nonreasonable expectations.

We performed tattooing in patients with stable corneal opacity and blind eyes with visual acuity of no light perception.

Informed consent was obtained before surgery after the aim of the procedure (cosmetic surgery with no hope for restoring vision), and possible complications were explained to patients with permission of photography pre- and postprocedure and use of photos for medical research. This study adhered to the tenets of the Declaration of Helsinki and was approved by the ethical committee of Sohag University.

The Rotring painting ink (Rotring Ink, Hamburg, Germany) (also known as China ink) was packed in a sterile glass infusion bottle for 20 minutes in a steam autoclave at 121°C. One of the authors (AHA) has previously demonstrated the safety and efficacy of using the same type of Rotring painting ink used in our study on male rabbits [12]. The stain color used was the black for all eyes.

**Figure 1:**
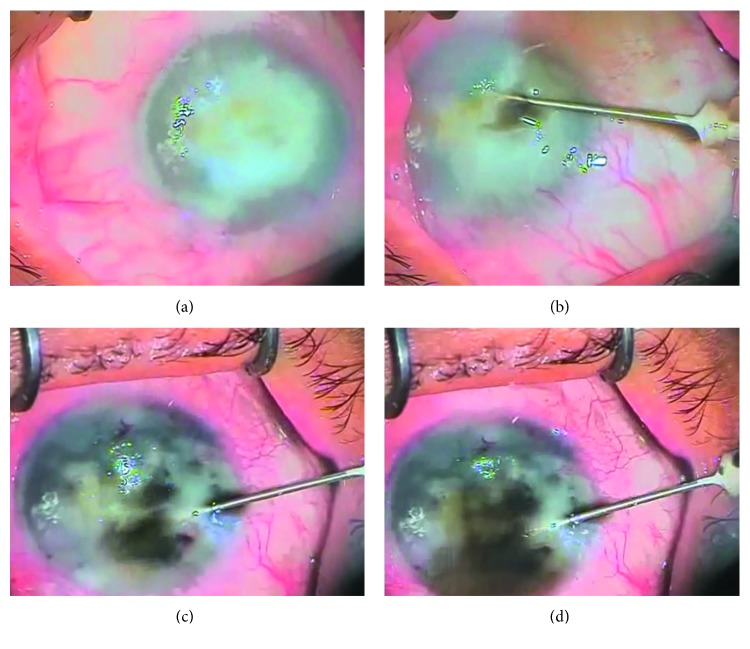
(a) Corneal opacification before tattooing. (b) The bevel of the needle was administered intrastromally tangential to the corneal surface, and injection was started. (c) Repeated multiple sites injections. (d) Full corneal tattooing.

The procedure was carried out in the operating room under sterile conditions by one surgeon (AHA) under topical anesthesia in all patients. Corneal epithelium was not removed. The ink was administered by multiple transepithelial intrastromal corneal injections using a 30-gauge needle attached to an insulin syringe with ink preloaded from a sterile cup. The bevel of the needle was up and administered tangential to the corneal surface to end up approximately in the midstroma avoiding accidental perforation of the cornea (Figures 1(a)–1(d), Supplementary Video 1). The number of injections was determined by the density of the scar and ranged from 4 to 8 injections. Saline solution was applied to irrigate the corneal surface to wash away excess ink and allow good visualization between injections. Contact lens was then applied and removed after 1 week. Postoperatively, 0.5% moxifloxacin hydrochloride and 1% prednisolone acetate eye drops were prescribed 5 times per day for 2 weeks. Ibuprofen 200 mg tablets were prescribed twice daily for 3 days. The patients were followed up at 1 day, 1 week, and 1, 3, 6, and 12 months. Photographs were taken after one month for comparison. Retreatment was done when needed as in inadequate coloration from the start or fading of the color.

## 3. Results

**Figure 2:**
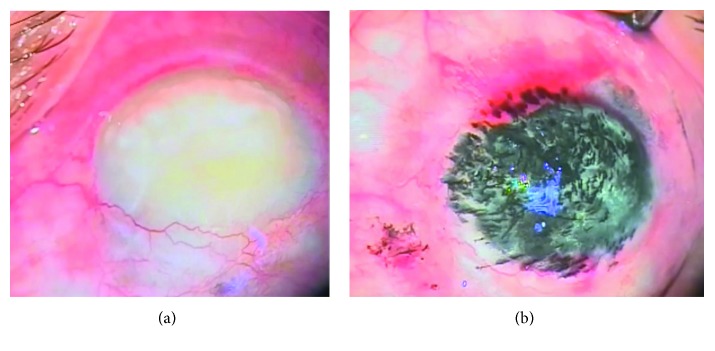
(a) Preoperative total corneal opacity and (b) Postoperative photo after corneal tattooing.

The patients enrolled in this study were between the age of 15 to 70. There were 29 females (55%) and 24 males (45%). Conjunctival injection, redness, and foreign body sensations were noticed in all patients in the early postoperative period that disappeared during the first week in all patients except one patient who developed epithelial defect which persisted for three weeks and resolved spontaneously by frequent lubricants and strong local antibiotics.  Figure 2 shows photos of before and after corneal tattooing of a corneal opacity.

**Figure 3:**
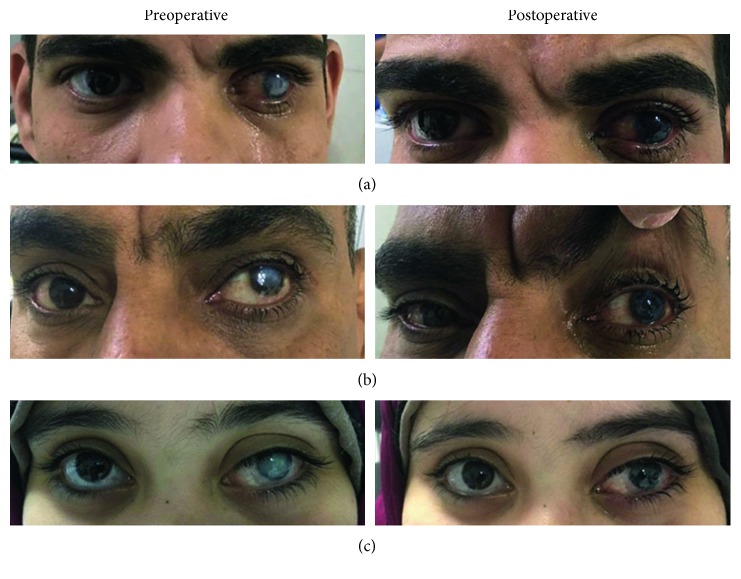
(a) Pretattooing and posttattooing (3 months) external pictures of a 32-year-old male. (b) Pretattooing and posttattooing (one month) external picture of a 62-year-old male. (c) Pretattooing and posttattooing (three months)external picture of a 18-year-old female.

**Figure 4:**
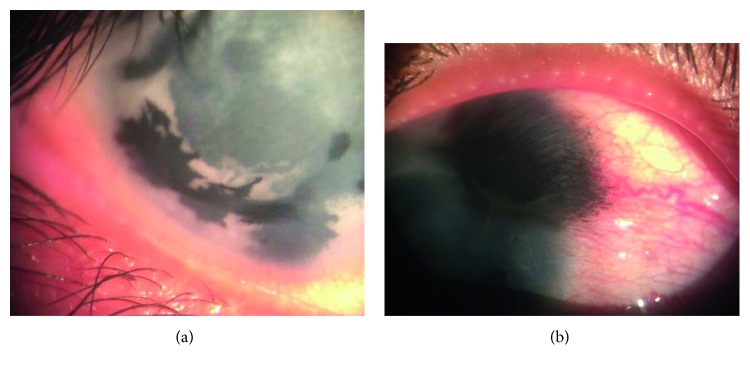
(a) Insufficient corneal staining. (b) Dye seepage into the conjunctiva.

Pre- and posttattooing external pictures of 3 patients from this study are shown in Figure 3. Insufficient corneal staining (Figure 4(a)) was detected in 3 eyes on the first postoperative week, and another session of corneal tattooing was carried out within the first postoperative month to tattoo the remaining opacity. Thus, most patients were satisfied about their general appearance.

There was one case of dye seepage into the conjunctiva as intraoperative complications which resolved gradually with time (Figure 4(b)). No corneal infections, epithelial erosions, or corneal melting were detected in any case along the follow-up period.

**Figure 5:**
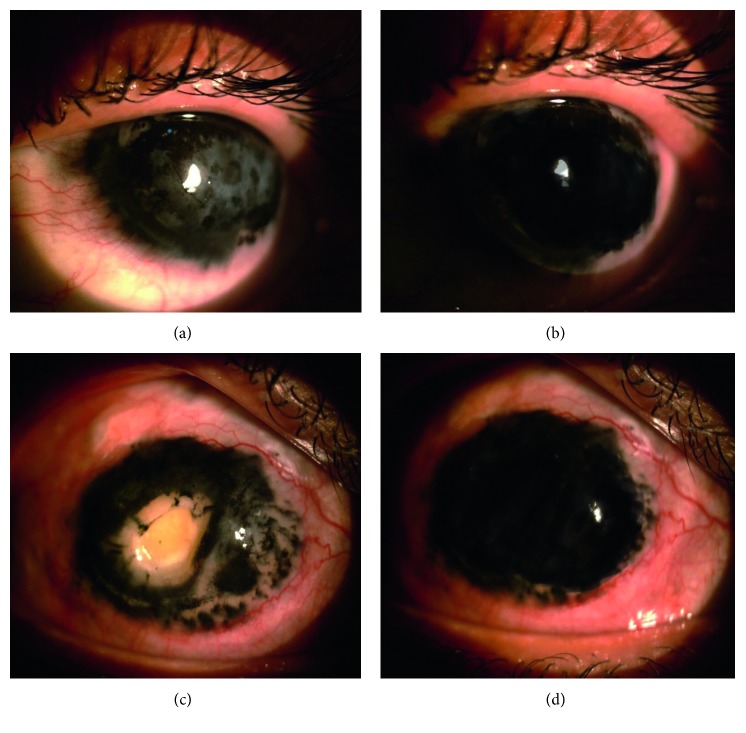
(a, c) Fading of the ink after 1 month; (b, d) retattooing after 3 months which made it homogeneous.

Late-onset complications after corneal tattooing included fading of color (3 eyes) or inconsistent dyeing of the opacity (3 eyes) (Figure 5). Both those complications were managed after 3 months by reinjection of the dye. The second intervention was appreciated by the patients. The total number of eyes that necessitated secondary injection was 9 eyes (17%). Patients' satisfaction after the end of the follow-up period was graded as unhappy or poor, happy or good, and very happy or excellent. Patients were asked to grade their satisfaction in numbers from 1 to 4: 1 is considered the poorest (unhappy), 2 is considered happy, 3 is considered very happy, and 4 is considered the best satisfaction result (excellent). The percentage of patient satisfaction was recorded in Table 2.

## 4. Discussion

Disfiguring corneal opacities may alter the personal self-confidence and affect badly both social lives and quality of life. Therefore, we should consider cosmetic repair as an essential line of treatment of corneal opacities in carefully selected cases to improve both the patient's self-confidence and social life [13].

Treatment options used in dealing with corneal opacity varied from keratoplasty to colored contact lenses and corneal tattooing. Keratoplasty is a very expensive procedure with many potential complications and so is usually restricted to patients with potential improvement of vision and low risk of rejection. Black pupil contact lenses were mostly used with smooth corneal surface in patients with no improvement of vision; however, there are many related complications especially in dusty hot atmospheres with poor hygienic conditions. So, corneal tattooing offers a viable option for patients who do not fit in the previous two options [14].

Various dyeing agents were reported in corneal tattooing such as India ink, organic colors, animal uveal pigment, China ink, and soot [1, 2]. The use of Rotring painting ink was favoured due to its availability, feasibility in storage, and sterilization due to its aqueous nature and economic price.

Inks are considered as stationery cosmetic products and offer an economic alternative to available classical corneal staining agents (metallic salts) [15].

Vassileva and Hristakieva, at Bulgaria universities, reported that the Indian ink is considered safe and long-lasting ink when properly diluted and it is widely used in corneal tattoo nowadays [16].

Several studies have concluded that corneal tattooing using china ink is considered durable with permanent effect as it is engulfed by keratocytes [8, 12] or endocytosed by corneal fibroblasts [17]. Some other theories proposed stability due to the fact that the cornea has no lymphatics, and thus, there is no diffusion or dispersion [18].

The safety of ink was evaluated in our study in which intrastromal corneal ink injection was found to be a safe technique of keratopigmentation with no side effects, and these results agree with a study of Amesty et al. [19] who concluded that intrastromal keratopigmentation technique reported good cosmetic results without adverse effects in the treated eyes.

We believe that injecting the ink intrastromally could add to the durability of the color and prevent its fading. There were many reports of introducing the ink intrastromally by either manually dissecting a lamellar corneal pocket [14] or using the Femtolaser to create a flap [10]. Intrastromal injection of the ink by multiple punctures by a syringe was our choice due to the nature of the corneal opacities (scarred and vascularized corneas) and to decrease the cost of the surgery.

Fading of colors and inconsistent dyeing of the opacity occurred in 6 eyes in our study postoperatively which necessitated reinjection of the dye. In a study of Kim et al. [14], they reported occurrence of reopacification and fading of color in 7 and 5 eyes, respectively, posttattooing which also necessitated retattooing like our cases with satisfactory results.

In conclusion, corneal tattooing with Rotring painting ink shows very favourable results with this simple and inexpensive procedure that moves corneal tattooing from an unattractive option for corneal opacities to a more renowned place in the line of management of disfiguring corneal opacity. However, longer follow-up period is recommended.

## Figures and Tables

**Table 1:** Causes of corneal opacity in recruited patients for corneal opacity.

Cause of corneal opacification	Eyes
Posttrauma	25
Postcorneal ulcer	20
Glaucoma	4
Unknown	4

**Table 2:** Percentage of patient satisfaction.

Grade of satisfaction	Eyes (%)
Excellent	32 (60)
Very happy	9 (17)
Happy	10 (19)
Unhappy	2 (4)

## Supplementary Materials

Supplementary Materials Supplementary Video 1: a video describes the technique of intrastromal corneal staining by Rotring painting Ink.
